# Supplementary material for: Exosome Tethering Requires Tetherin Homodimerisation
Source: Biol Cell. 2025 Dec 18;117(12):e70046. doi: 10.1111/boc.70046 (PMC12712889; doi:10.1111/boc.70046)

Supplementary Figure 1

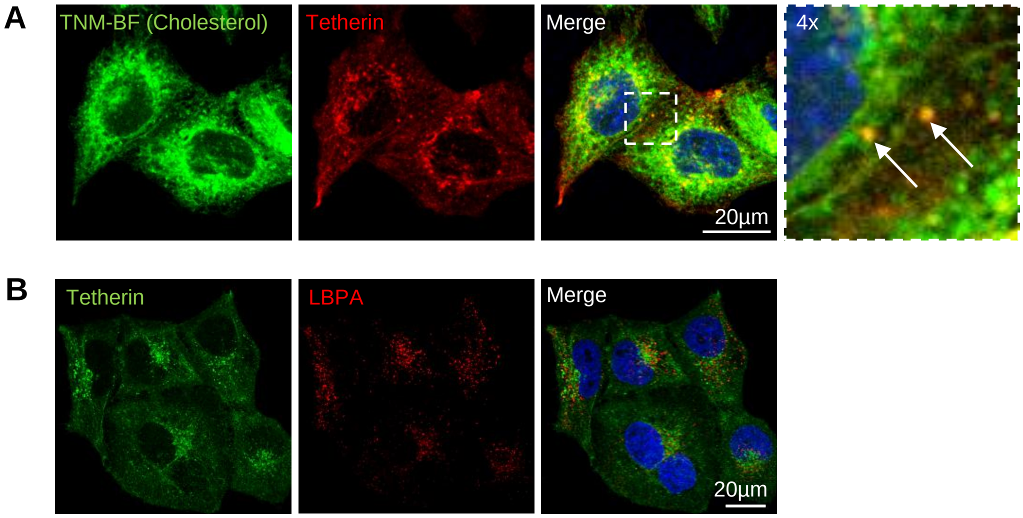

Supplementary Figure 2

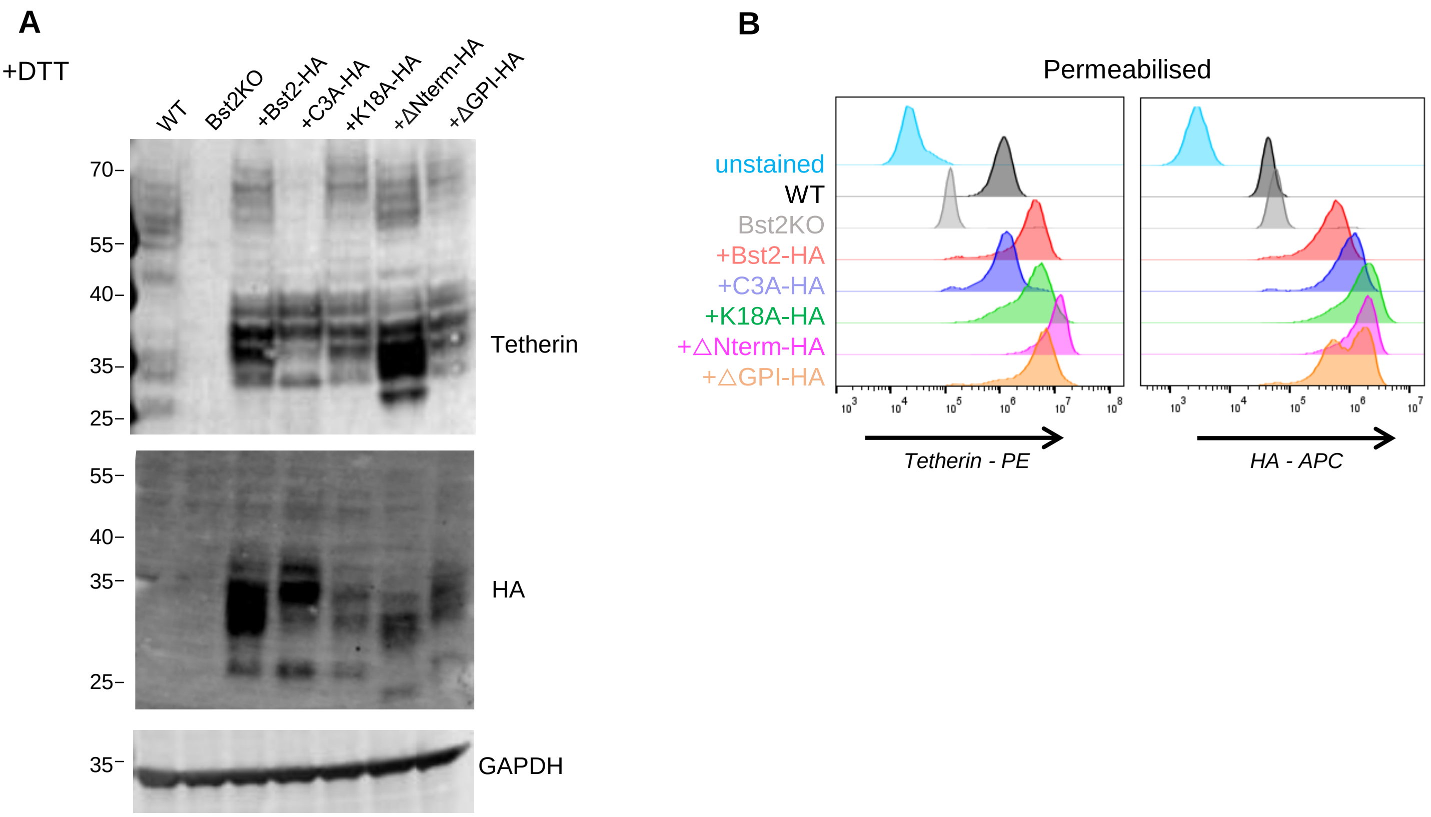

Supplementary Figure 3

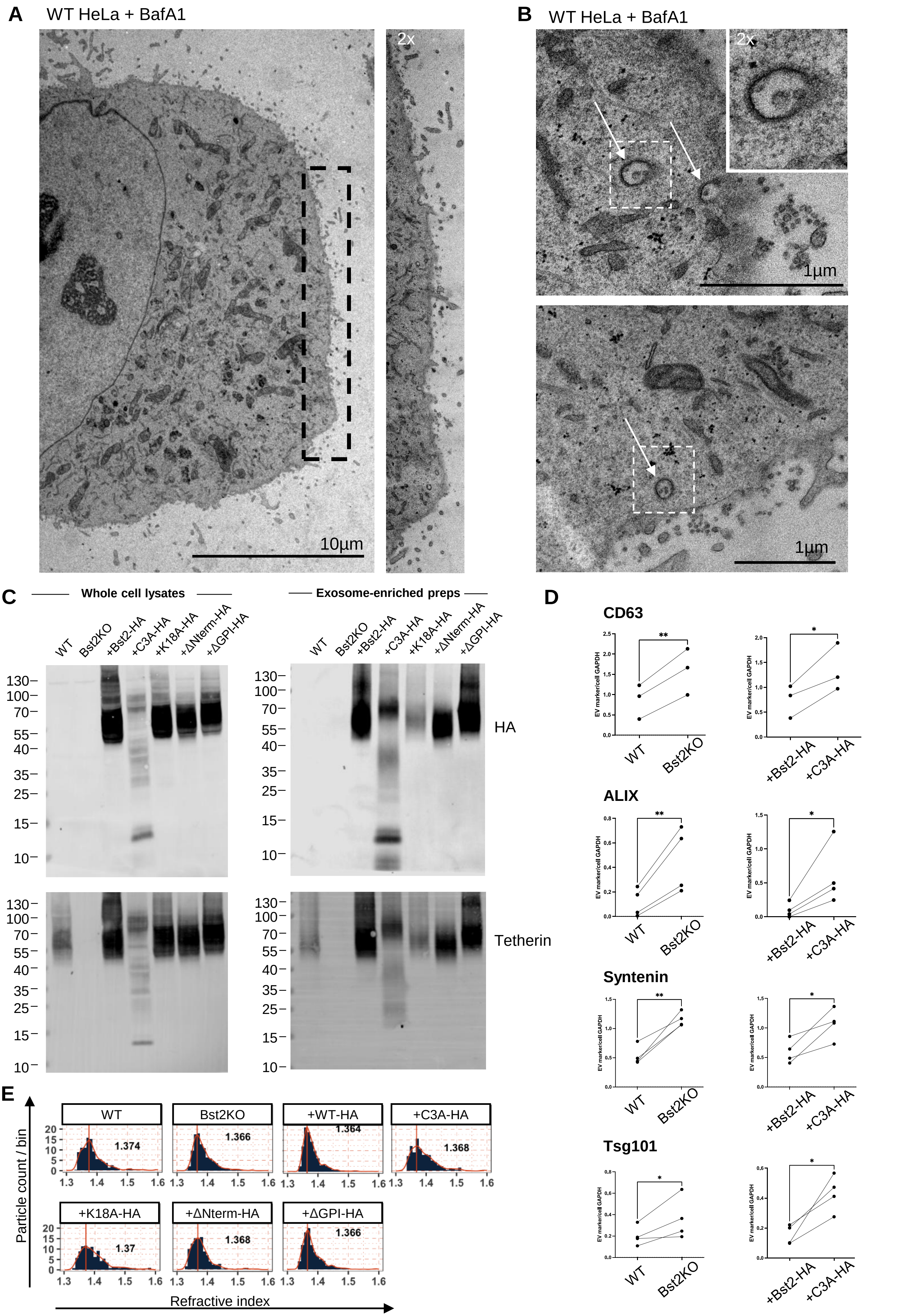

Supplementary Figure 4

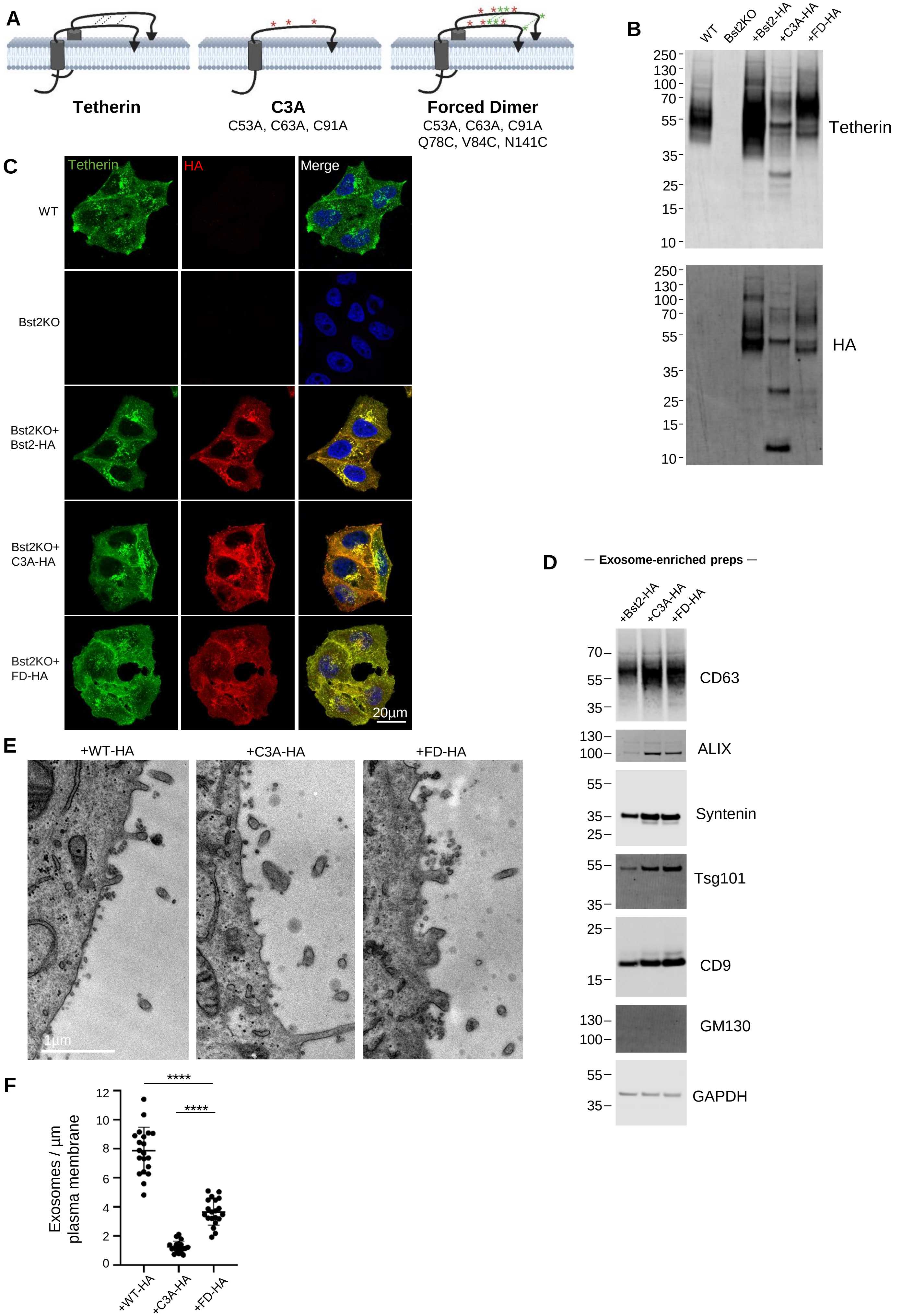

Supplementary Figure 5

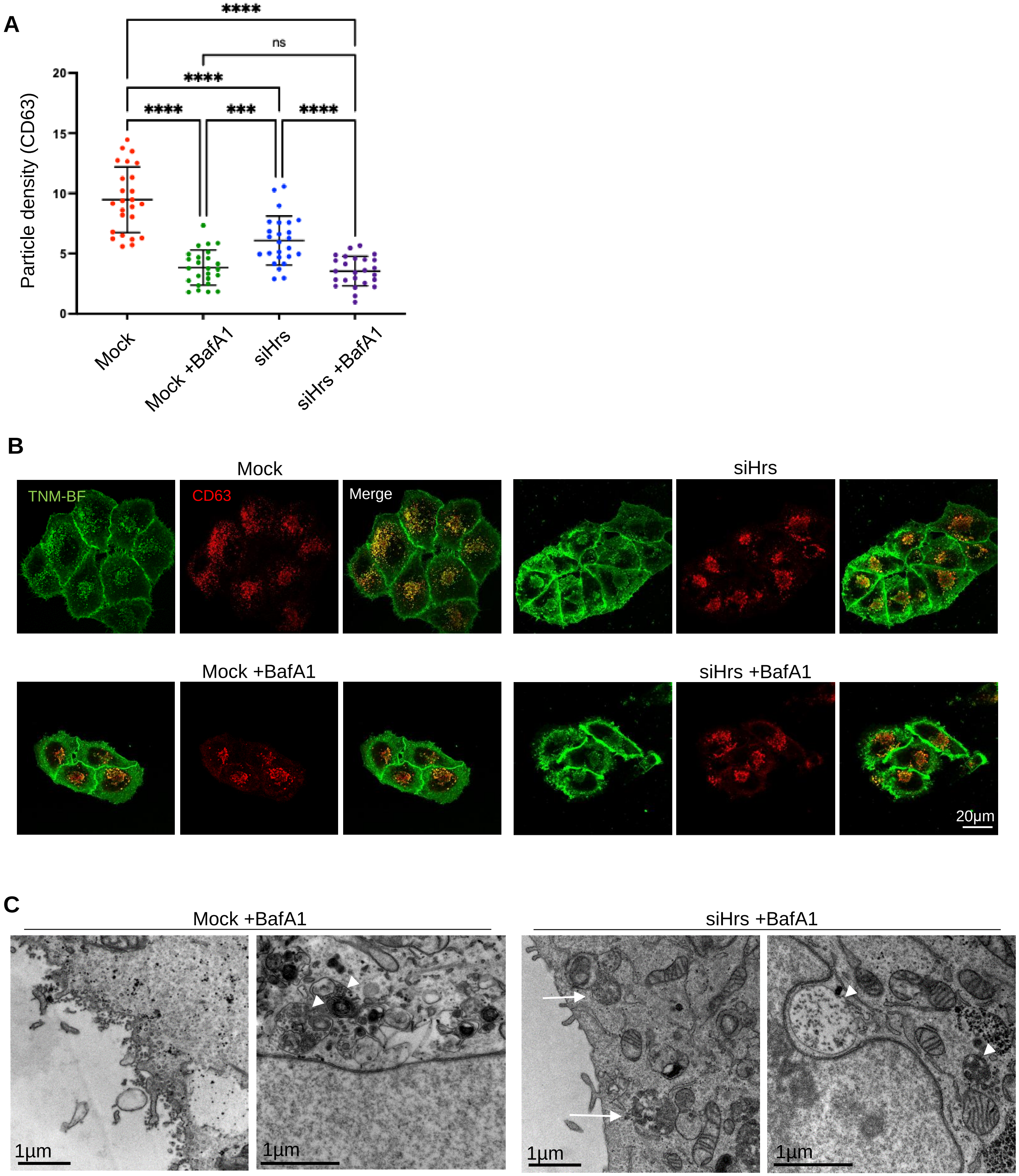

Supplementary Figure 6

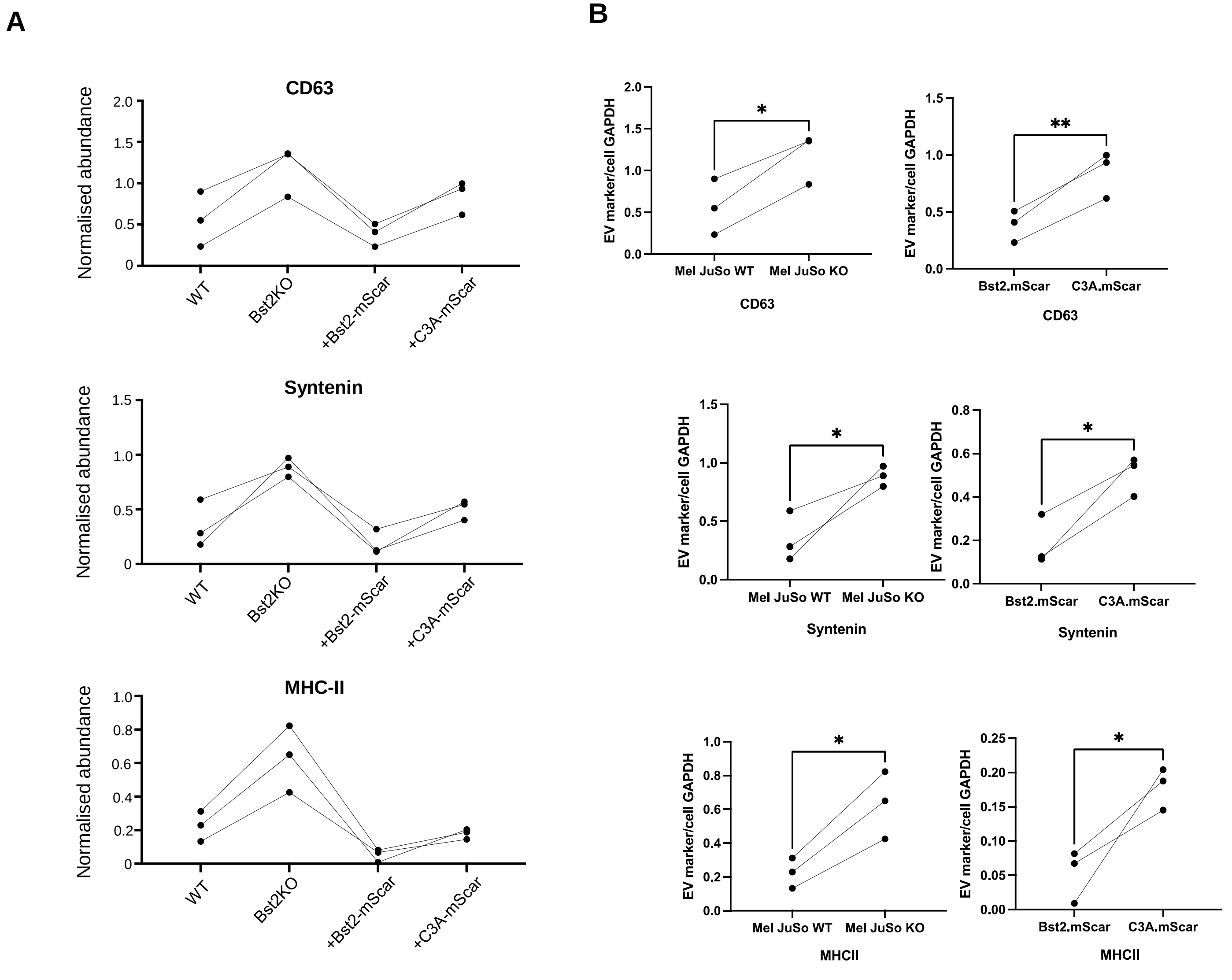

Supplementary Figure 7

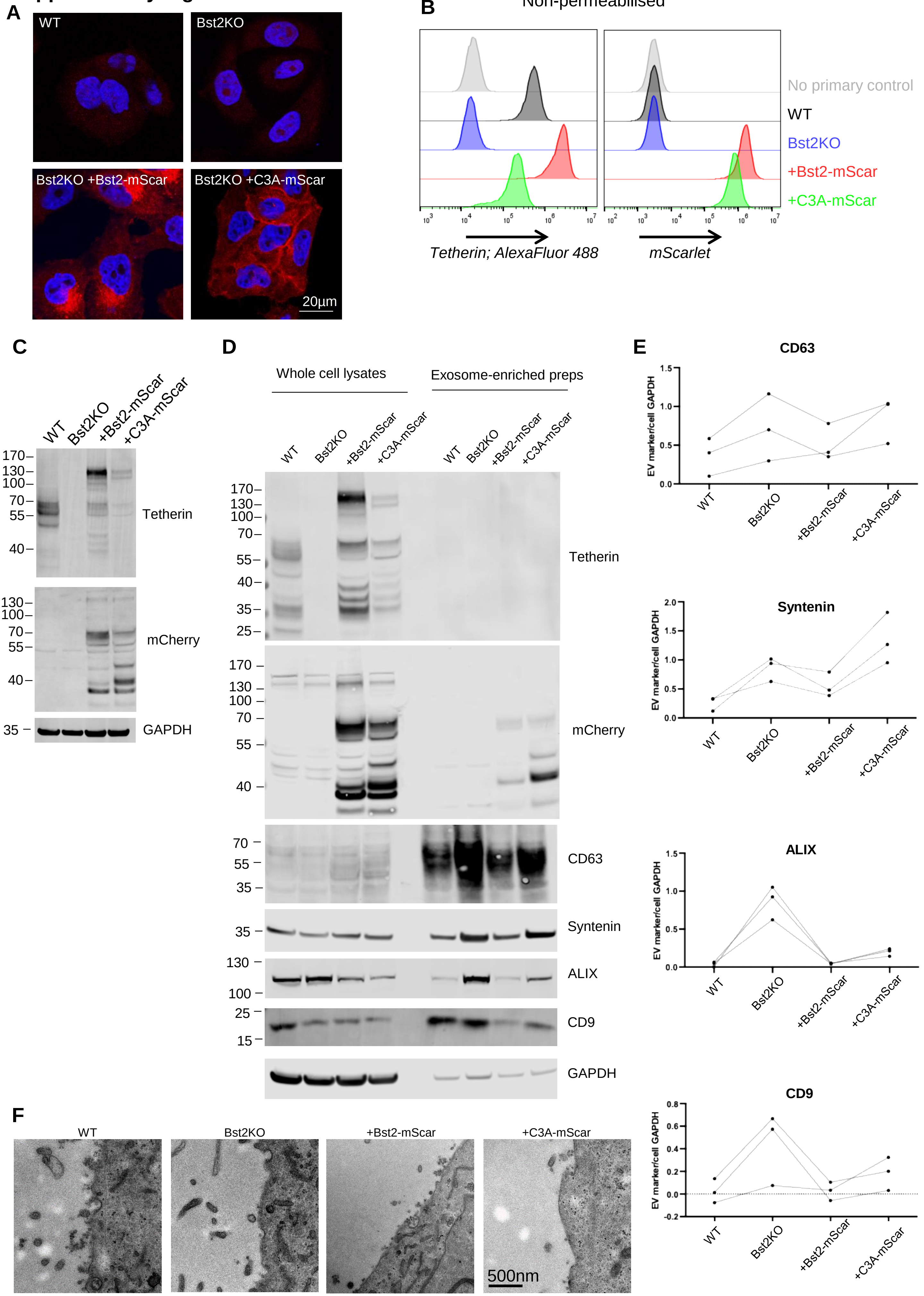

Supplement: Supplementary file 1 — Supporting Information File 1: boc70046‐sup‐0001‐SuppMat.pdf [file BOC-117-e70046-s001.pdf]
